# Noncompliance to guidelines in head and neck cancer treatment; associated factors for both patient and physician

**DOI:** 10.1186/s12885-015-1523-3

**Published:** 2015-07-11

**Authors:** Emilie A. C. Dronkers, Steven W. Mes, Marjan H. Wieringa, Marc P. van der Schroeff, Robert J. Baatenburg de Jong

**Affiliations:** Department of Otorhinolaryngology and Head and Neck Surgery, Erasmus University Medical Center, ‘s Gravendijkwal 230, room D112, 3015 CE Rotterdam, The Netherlands

**Keywords:** Head and neck cancer, Nonstandard treatment, Patient compliance, Survival

## Abstract

**Background:**

Decisions on head and neck squamous cell carcinoma (HNSCC) treatment are widely recognized as being difficult, due to high morbidity, often involving vital functions. Some patients may therefore decline standard, curative treatment. In addition doctors may propose alternative, nonstandard treatments. Little attention is devoted, both in literature and in daily practice, to understanding why and when HNSCC patients or their physicians decline standard, curative treatment modalities. Our objective is to determine factors associated with noncompliance in head and neck cancer treatment for both patients and physicians and to assess the influence of patient compliance on prognosis.

**Methods:**

We did a retrospective study based on the medical records of 829 patients with primary HNSCC, who were eligible for curative treatment and referred to our hospital between 2010 and 2012. We analyzed treatment choice and reasons for nonstandard treatment decisions, survival, age, gender, social network, tumor site, cTNM classification, and comorbidity (ACE27). Multivariate analysis using logistic regression methods was performed to determine predictive factors associated with non-standard treatment following physician or patient decision. To gain insight in survival of the different groups of patients, we applied a Cox regression analysis. After checking the proportional hazards assumption for each variable, we adjusted the survival analysis for gender, age, tumor site, tumor stage, comorbidity and a history of having a prior tumor.

**Results:**

17 % of all patients with a primary HNSCC did not receive standard curative treatment, either due to nonstandard treatment advice (10 %) or due to the patient choosing an alternative (7 %). A further 3 % of all patients refused any type of therapy, even though they were considered eligible for curative treatment. Elderliness, single marital status, female gender, high tumor stage and severe comorbidity are predictive factors. Patients declining standard treatment have a lower overall 3-year survival (34 % vs. 70 %).

**Conclusions:**

Predictive factors for nonstandard treatment decisions in head and neck cancer treatment differed between the treating physician and the patient. Patients who received nonstandard treatment had a lower overall 3-year survival. These findings should be taken into account when counselling patients in whom nonstandard treatment is considered.

## Background

Decisions concerning cancer treatment are becoming more complex. On the one hand, there is a strong tendency to apply standards and guidelines. On the other hand, cancer patients are considered partners in decision making in order to incorporate individual perspectives and needs. Moreover, patients are better informed about treatment options than they used to be. The fine balance between benefits and side-effects of treatment is increasingly presented and discussed with the patient in an informed or shared decision making process. Still, the use of guidelines is advocated to assure optimal treatment proposals for similar patients.

It is known that a proportion of cancer patients does not receive standard, guideline driven, treatment for cancer that could be curatively treated, either by choice of their physician or by their own choice. Yet, little is known about this specific, non-compliant patient population. How frequently does it occur that patients themselves refuse standard therapy for cancer, even if they are considered eligible for curative treatment by their physician, and what are the reasons for this behavior? This question is particularly interesting if survival rates are low and treatments are associated with morbidity and mortality as well.

Head and neck squamous cell carcinoma (HNSCC) describe a range of squamous cell tumors that arise from the head and neck region, which includes the oral cavity, pharynx, larynx and nasal cavity. The worldwide incidence of head and neck cancer exceeds half a million cases annually, ranking it as the fifth most common cancer worldwide [1, 2]. Five year survival rates for cancers in the head and neck area are about 50 % [1]. In the majority of cases, treatment consists of surgery, radiotherapy, chemotherapy and combinations of these modalities. All types of treatment are associated with high morbidity, sometimes compromising vital functions, including respiration, swallowing and speech, and have an enormous impact on the quality of life. Therefore, improved cure rate may come at the price of increased short-term and long-term morbidity and decreased quality of life. Cure is not always the main priority for the head and neck cancer patient. For example, up to 20 % of patients would accept a lesser chance of cure to avoid a laryngectomy and to keep their normal voice [3, 4]. Hence, decisions on head and neck cancer treatment are widely recognized as being difficult [5, 6].

Our primary objective is to determine frequencies of and predictors for receiving a nonstandard treatment in HNSCC and to explore reasons for choosing a nonstandard treatment, either by patients or physicians. As a secondary objective we want to assess the influence on prognosis of receiving nonstandard treatment for curative HNSCC.

## Methods

### Subjects

This retrospective study, based on medical records, included patients with newly diagnosed HNSCC without distant metastasis. Patients with cancer of the lip, oral cavity, nasopharynx, oropharynx, hypopharynx, and larynx which could be treated with curative intent qualified for this study. Recurrent or residual cancer was excluded but patients with second primary HNSCC were deemed eligible. Patients who were enrolled in any clinical trial in this period were also excluded. In the period from January 2010 to December 2012, 829 patients were included. The study was carried out in compliance with the Helsinki declaration and was approved by the ethics committee of the Erasmus Medical Center, including a waiver for informed consent.

All patients were initially set for curative treatment at the Erasmus Medical Center Rotterdam, the Netherlands. The tumor stage at the time of first diagnosis was classified according to the clinical staging system described by the Union for International Cancer Control (UICC). A first treatment proposal was presented at the regional multidisciplinary head and neck tumor conference, where all new patients were discussed. The multidisciplinary tumor board (MDT) consisted of oncologists, head and neck surgeons, and radiotherapists. The treatment proposal was weighed up against the standard treatment protocol, which is based on national guidelines published by the Comprehensive Cancer Centre the Netherlands (IKNL) and regional additions. The final proposal may be according to the guidelines (standard treatment) or deviant (nonstandard treatment). Reasons for nonstandard treatment, either as a result of MDT or patient decision, were collected retrospectively. Solely major deviations of standard guidelines were marked as ‘nonstandard’ treatment. A change in dose of radiotherapy or chemotherapy was not accepted as a deviation of standard guidelines, but refusing total laryngectomy indeed was.

### Outcomes

Following the discussion in the MDT, the treatment proposal was discussed with the patient. In the decision making process, patients may have either accepted or declined the proposal. In this study, we considered the following groups.

1. Standard treatment according to guidelines (reference group)
2. Nonstandard treatment as proposed by the multidisciplinary tumor board
3. Nonstandard treatment as desired by the patient:
  - Alternative (less extensive)
  - No treatment at all

Different parameters present at the time of diagnosis, were retrospectively collected for every patient. These included age at diagnosis, year of diagnosis, tumor site, tumor stage**,** gender, marital status, having children, comorbidity conditions, prior malignancy (head and neck or other), treating physician (head and neck oncologist, radiotherapist or general oncologist) and survival. The presence of one or more different comorbid ailments was coded for all patients using Adult Comorbidity Evaluation-27 (ACE-27) [7]. The ACE-27 grades specific comorbid conditions in different organ systems into one of three levels of comorbidity. The overall comorbid score is graded in four levels, none, mild, moderate or severe and is based on the highest ranked single ailment. Patients with two or more moderate ailments in different organ systems or disease groupings are graded as severe. The ACE-27 is a comprehensive tool, commonly used in head and neck cancer literature, and accurate as a retrospective measuring instrument of comorbidity.

The retrospective analysis of the specified characteristics was performed by the first two authors (EACD an SWM) who were not involved in decision making by the multidisciplinary tumor team.

### Statistical analysis

The data was analyzed with IBM SPSS Statistics version 21.0 for Windows. For statistical processing, several variables were converted to dichotomous values, based on experience, evidence from literature, or distribution of data following a normal Gaussian curve with a cutoff point at the mean. This was the case for age (<65 or ≥65 years), marital status (partner or single), comorbidity (ACE-score 0–1 or ACE-score 2–3), tumor site (pharynx, larynx and oral cavity) and tumor stage (stage I-II or stage III-IV). Descriptive statistics,χ^2^ tests and simple logistic regression methods were used to compare three groups (reference group, nonstandard treatment by MDT decision and nonstandard treatment by patient’s decision) P-values <0.05 were considered to be statistically significant.

Multivariate analysis using logistic regression methods and taking into account interaction terms was performed to determine predictive factors associated with non-standard treatment following MDT or patient decision [8]. A predictor was defined as a predictive factor that contributes independently and significantly (p-value of < 0.05) to the choice of non-standard treatment, done either by the MDT or by patient decision. In general, the limiting sample size in logistic regression analysis is the number of events of interest. The assumption is made that this analysis will produce reasonably stable estimates of the effect of each variable on the outcome if the limiting sample size allows a ratio of approximately 10 to 15 observations per possible predictive factor [8]. To gain insight into the impact of each possible predictor in the model, all variables were entered in the logistic regression analysis at the same time. The following factors were included: age at diagnosis, year of diagnosis, gender, marital status, having children, tumor stage, tumor site , comorbidity, prior malignancy and prior head and neck malignancy, and type of initial treatment following national guidelines. Stratification by gender was done following the analysis for interaction terms. To design a final stratified model showing independently and significantly predictive factors associated with non-standard treatment following MDT or patient decisions, a backward selection procedure was applied, accepting predictors with a p-value <0.05. Following this, a forward selection procedure was done to confirm our results.

To gain insight in survival of the different groups of patients, we applied a Cox regression analysis. After checking the proportional hazards assumption for each variable, we adjusted the survival analysis for gender, age, tumor site, tumor stage, comorbidity and a history of having a prior tumor.

## Results

The demographics of all included patients and the demographics of the distinguished subgroups of patients are listed in Table 1. 82.9 % (*n = 687)* of patients received treatment according to guidelines. The remaining 17.1 % (*n* = 142) received nonstandard treatment or no treatment at all. Deviation from protocol in these patients was motivated. In 10.7 % (*n* = 89) of all patients the multidisciplinary team decided to propose a nonstandard treatment. The mean age of these patients was 67 years at the time of diagnosis and 22 % of them were female. As shown in Table 2 levels of comorbidity, stage of disease, tumor site, initial treatment proposal and marital status differed significantly between this group and the patients who received standard treatment. In multivariate logistic regression analysis many of these characteristics were significantly associated with the outcome of nonstandard tumor board advice. These characteristics are marked by an asterisk in Table 2. A proportion of 7.2 % (*n* = 60) of all patients declined a standard treatment proposal given by the multidisciplinary team. The mean age of this group of patients at the time of diagnosis was 72 years and 47 % of them was female, whereas the proportion of female subjects of the total population was just 28 %. In 4.2 % (*n* = 35) of all patients, a part of the treatment was refused by patients themselves and as a result they received less extensive therapy. A further 3 % (*n* = 25) of all patients refused any type of therapy, despite being considered eligible for curative treatment by the multidisciplinary team. Gender, age, levels of comorbidity, stage of disease and marital status differed between patients who received standard treatment and those who chose nonstandard treatment against the advice of the MDT (Table 3). Multivariate logistic regression analysis showed that several of these variables were significantly associated with the outcome of patients declining or refusing standard treatment. Following the outcomes, stratification by gender was done to specify the influence of the other variables between men and women on decisional behavior. In the group of females who declined standard curative treatment, being older than 65 years at time of diagnosis and being single or widowed were significant predictors. On the other hand, only advanced tumor stage was a significant predictor in male patients who declined standard curative treatment.

**Table 1:** Demographic characteristics of total population and distinguished subgroups

		Nonstandard treatment (*N* = 142)*	
	Total population (*N* = 829)	Proposed by the MDT (*N* = 89)	Desired by the patient (*N* = 60)
Age (years) (mean and standard deviation)	63.9 (11.1)	67.2 (10.9)	71.2 (12.2)
Gender			
*Male*	596 (72 %)	69 (78 %)	32 (53 %)
*Female*	233 (28 %)	20 (22 %)	28 (47 %)
Comorbidity score (ACE-27)			
*0*	182 (22 %)	3 (3 %)	11 (18 %)
*1*	327 (39 %)	30 (34 %)	18 (30 %)
*2*	239 (29 %)	36 (40 %)	22 (37 %)
*3*	81 (10 %)	20 (23 %)	9 (15 %)
Tumor stage			
*1*	162 (20 %)	5 (6 %)	8 (13 %)
*2*	180 (22 %)	6 (7 %)	7 (12 %)
*3*	161 (19 %)	26 (29 %)	7 (12 %)
*4*	326 (39 %)	52 (58 %)	38 (63 %)
Tumor site			
*Lip*	24 (3 %)	0 (0 %)	1 (1 %)
*Nasopharynx*	29 (4 %)	4 (4 %)	25 (42 %)
*Oral cavity*	255 (31 %)	17 (19 %)	17 (29 %)
*Oropharynx*	213 (26 %)	27 (30 %)	6 (10 %)
*Supraglottic larynx*	89 (11 %)	13 (15 %)	3 (5 %)
*Glottic larynx*	124 (15 %)	8 (9 %)	8 (13 %)
*Hypopharynx*	95 (11 %)	20 (23 %)	0 (0 %)
Prior malignancy			
*No*	669 (81 %)	70 (79 %)	47 (78 %)
*Other prior cancer yet treated*	84 (10 %)	7 (8 %)	8 (13 %)
*Prior head and neck cancer yet treated*	76 (9 %)	12 (13 %)	5 (9 %)
Marital status			
*Partner*	569 (69 %)	50 (56 %)	27 (45 %)
*Single*	260 (31 %)	39 (44 %)	33 (55 %)
Standard treatment according to guidelines			
*Radiotherapy*	185 (22 %)	10 (11 %)	4 (7 %)
*Chemoradiation*	183 (22 %)	47 (53 %)	14 (23 %)
*Surgery + radiotherapy*	208 (25 %)	20 (22 %)	29 (48 %)
*Surgery + chemotherapy*	12 (1 %)	3 (3 %)	0 (0 %)
*Surgery + postoperative radiation (PORT) on indication*	116 (14 %)	4 (5 %)	7 (12 %)
*Surgery*	125 (15 %)	5 (6 %)	6 (10 %)
Year of treatment			
*2010*	234 (28 %)	27 (30 %)	21 (35 %)
*2011*	259 (31 %)	22 (25 %)	21 (35 %)
*2012*	335 (41 %)	40 (45 %)	18 (30 %)

*In seven patients, both MDT and patient were non-compliant to standard treatment guidelines; patients received a proposal of nonstandard treatment by the MDT but however refused any treatment

**Table 2:** Unadjusted and adjusted OR’s for MDT decision to propose nonstandard treatment

Characteristic		*OR unadjusted*	95 % CI	*OR adjusted* ^*b*^	95 % CI
Age	<65 years ^a^	1.22	0.7–1.9	1.46	0.9–2.4
	≥65 years				
Gender	Male ^a^	0.72	0.4–1.2	0.72	0.4–1.3
	Female				
Comorbidity score (ACE-27)	Low (0–1) ^a^	3.06*	1.9–4.8	3.40*	2.0–5.7
	High (2–3)				
Tumor stage	Early (I-II) ^a^	5.74*	3.0–11.0	3.40*	1.4–8.5
	Advanced (III-IV)				
Tumor site	Oral cavity ^a^				
	Pharynx	2.75*	1.6–4.9	0.94	0.4–2.2
	Larynx	1.69	0.9–3.3	0.85	0.3–2.1
Prior malignancy	No ^a^				
	Other prior cancer yet treated	0.78	0.3–1.6	0.61	0.3–1.5
	Prior head and neck cancer yet treated	1.60	0.8–3.1	2.56*	1.1–5.7
Marital status	Partner ^a^	1.83*	1.2–2.9	1.68	1.0–2.9
	Single				
Children	Yes ^a^				
	No	1.23	0.7–2.1	1.07	0.6–2.0
	Unknown	0.79	0.4–1.5	1.31	0.7–2.7
	No contact	0.84	0.7–1.1	0.90	0.7–1.1
Standard treatment according to guidelines	Surgery ^a^				
	Radiotherapy	1.37	0.5–4.1	1.31	0.4–4.5
	Chemoradiation	8.29*	3.2–21.5	4.79*	1.3–17.1
	Surgery + radiotherapy	2.53	0.9–7.0	1.13	0.3–4.0
	Surgery + chemotherapy	8.00*	1.6–39.0	4.61	0.7–29.5
	Surgery + PORT on indication	0.86	0.2–3.3	0.98	0.2–4.2
Year of treatment	2010 ^a^				
	2011	0.71	0.4–1.3	0.56	0.3–1.1
	2012	1.04	0.6–1.7	1.11	0.6 2.0

^a^ = reference value, ^b^ = odds ratio calculated using multivariate logistic regression analysis adjusting for age, gender, comorbidity, tumor stage, tumor site, prior malignancy, marital status, having children, standard treatment proposal according to guidelines, year of treatment, * = *p* value < 0.05

**Table 3:** Unadjusted and adjusted OR’s for patient decisions to choose nonstandard treatment and adjusted OR’s after stratification for gender

		Not stratified analysis				Stratification for gender			
Characteristic		*OR unadjusted*	95 % CI	*OR adjusted* ^*b*^	95 % CI	*♂OR adjusted* ^*c*^	95 % CI	*♀OR adjusted* ^*c*^	95 % CI
Age	<65 years ^a^	3.19*	1.8–5.7	3.40*	1.8–6.4	1.73	0.8–3.6	7.22*	2.4–22.1
	≥65 years								
Gender	Male ^a^	2.41*	1.4–4.1	2.66*	1.5–4.8	-	-	-	-
	Female								
Comorbidity score (ACE-27)	Low (0–1) ^a^	1.78*	1.0–3.0	1.49	0.8–2.7	1.77	0.8–3.7	1.39	0.6–3.3
	High (2–3)								
Tumor stage	Early (I-II) ^a^	2.22*	1.2–4.1	1.07	0.4–2.9	2.88*	1.2–7.1	1.92	0.8–4.7
	Advanced (III-IV)								
Tumor site	Oral cavity ^a^					-	-	-	-
	Pharynx	0.78	0.4–1.4	1.34	0.6–3.0				
	Larynx	0.43*	0.2–0.9	0.82	0.3–2.1				
Prior malignancy	No ^a^					-	-	-	-
	Other prior cancer yet treated	1.39	0.6–3.1	1.09	0.5–2.6				
	Prior head and neck cancer yet treated	0.93	0.4–2.4	0.99	0.3–2.9				
Marital status	Partner ^a^	2.92*	1.7–5.0	2.25*	1.2–4.1	1.64	0.8–3.5	3.63*	1.5–9.0
	Single								
Children	Yes ^a^					-	-	-	-
	No	2.26*	1.3–4.1	2.08*	1.0–4.1				
	Unknown	0.78	0.3–1.8	1.19	0.5 3.0				
	No contact	1.66	0.4–7.5	1.19	0.2–5.9				
Standard treatment according to guidelines	Surgery ^a^					-	-	-	-
	Radiotherapy	0.44	0.1–1.6	0.29	0.07–1.2				
	Chemoradiation	1.64	0.6–4.4	1.15	0.3–4.6				
	Surgery + radiotherapy	3.21*	1.3–8.0	2.16	0.6–7.6				
	Surgery + chemotherapy	-	-	-	-				
	Surgery + PORT on indication	1.27	0.4–3.9	1.10	0.3–3.8				
Year of treatment	2010 ^a^					-	-	-	-
	2011	0.89	0.5–1.7	0.73	0.4–1.5				
	2012	0.57	0.3–1.1	0.63	0.3–1.3				

^a^ = reference value, ^b^ = odds ratio calculated using multivariate logistic regression analysis adjusting for age, gender, comorbidity, tumor stage, tumor site, prior malignancy, marital status, having children, standard treatment proposal according to guidelines, year of treatment, ^c^ = odds ratio stratified for gender (male versus female) calculated using multivariate logistic regression analysis adjusting for age, comorbidity, tumor stage and marital status, * = *p* value < 0.05

Solely major deviations from standard treatment guidelines were accepted as being ‘nonstandard’ treatment. Table 4 shows the various reasons the MDT gave for not recommending a standard, guideline-driven treatment for 10 % of all patients included in this study. Reasons put forward by 7 % of patients declining standard treatment are also shown in Table 4. These patients were all considered eligible for curative treatment, however, chose not to follow proposals of the MDT. In most cases, patients didn’t want an extensive type of treatment which would have a great impact on their lives. When the MDT decided to advise a nonstandard therapy their arguments were more about poor physical conditions of the patients, for example cardiovascular disease or insufficient kidney function.

**Table 4:** Reported reasons of MDT members for not recommending guideline-driven treatment and reported reasons of patients for refusing standard curative treatment proposed by their physician

**Reported reasons of MDT members**	Number of cases	Percentage
No surgery because of patient conditions	18	20 %
No chemotherapy because of patient conditions	28	32 %
No radiotherapy because of patient conditions	6	7 %
No treatment because of patient conditions	5	6 %
No radiotherapy because of medical history	4	4 %
Customized chemotherapy because of patient conditions	20	22 %
Customized radiotherapy because of patient conditions	5	6 %
Customized surgery because of patient conditions	1	1 %
Customized therapy because of patient conditions	2	2 %
*Total*	*89*	*100 %*
**Reported reasons of patients**	Number of cases	Percentage
Patient declines any treatment	11	18 %
Patient declines surgery	19	32 %
Patient declines radiotherapy	12	20 %
Patient declines chemotherapy	4	7 %
Patient cannot mentally handle therapy	11	18 %
Patient declines therapy because of GP recommendation	2	3 %
Patient declines therapy because of religious beliefs	1	2 %
*Total*	*60*	*100 %*

### Survival

Following nonstandard or even non-curative treatment one can imagine that survival will be worse in these patients. Still, it is relevant to know to which extent survival will drop in these patients.

**Fig. 1:**
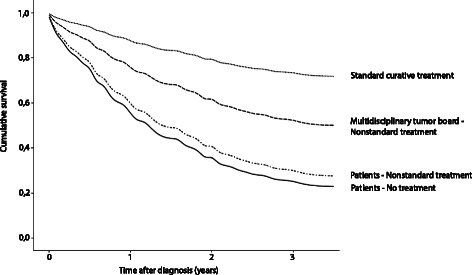
Cumulative estimated survival rates per year for 4 distinguished patient groups adjusted for gender, age, tumor site, tumor stage, comorbidity and a history of having a prior tumor

Patients who received nonstandard treatment had a significantly lower overall 3-year survival (34 % vs. 70 %). Survival for patients who received nonstandard treatment due to a decision made by the multidisciplinary team was decreased (HR 2.1 (1.49–3.03), *p* < 0.001). Survival decreased even more in patients who declined standard treatment themselves (HR 3.9 (2.34–6.31), *p* < 0.001) or refused any type of treatment (HR 4.5 (2.72–7.31), *p* < 0.001). For illustrative purposes we made four separate lines in Fig. 1, using the cumulative estimated survival rates per month, calculated with the adjusted Cox regression analysis. These lines represent four categories of patients: those who receive standard curative treatment, those who receive nonstandard treatment due to a decision by the MDT, those who wish for a less extensive though nonstandard type of treatment and those who reject any type of treatment.

## Discussion

One of the major topics in oncology today is to strive for personalized medicine. Decisions on cancer treatment are complex regarding guidelines on the one hand and patients preferences on the other hand. This specifically holds true if survival rates are relatively poor and treatments are associated with morbidity and mortality, as is the case in HNSCC. Counselling of patients and informed decision making is important, and as a result, a proportion of patients will not receive standard curative treatment. Doctors are generally not aware of the extent of this situation. Our study shows that 17 % of all patients with a primary HNSCC did not receive standard curative treatment, either due to a nonstandard treatment advice, or due to the patient choosing an alternative. The MDT decided in 10 % of all patients to advise nonstandard treatment in the case of a primary and curable HNSCC. Seven percent of all patients decided themselves to decline standard curative treatment advice. A proportion of 4 % wished for a less extensive type of treatment and 3 % refused any type of therapy. Reflecting on the various reasons mentioned for choosing a nonstandard treatment for curative HNSCC, there is a difference in argumentation between patients and physicians. Physicians focused more on physical aspects, essentially comorbidity and advanced disease, whereas decisions of patients were based on quality of life and emotional or psychological reasons. We should look at these results with some caution because a retrospective chart review is not an optimal way of identifying reasons of patients to refuse treatment. Patient surveys or interviews appear to be more efficient [9].

A review of literature on head and neck cancer showed three other studies focusing partly on our objectives [10–12]. In agreement with these studies, we found that a higher comorbidity index and poor physical functioning were associated with nonstandard treatment. Parallel to our results, social factors were also predictive for nonstandard treatment, as widowed persons were more often not treated according to the standard protocol. [10] Still, there were some major differences in methodology between the studied articles and our study. One study did not perform a multivariable analysis and therefore did not adjust for the influence of other predictive factors [11]. In this study patients with recurrent or residual disease were also included. Another study excluded patients with a low tumor stage and patients aged between 60 and 70 years [10]. The last study included only elderly patients [12]. A limitation of our own study would be its retrospective nature, which may have led to some information bias since not all data on the social network of our patients was available. Also, this study was performed in one large center in the Netherlands, and therefore it could be less generalizable for an international population. On the other hand, although national guidelines on head and neck cancer treatment may differ between countries regarding dosages of radiotherapy or details in surgical techniques, the assumption can be made that explicit major deviations of guidelines are comparable. And therefore our results could be applied to an international population of head and neck cancer patients. When comparing our results to previous studies on this subject done in general oncology, there are certain similarities. Various factors claimed to be associated with cancer treatment refusal include: lower social class, higher education, single or divorced, patients living in a rural community, older age group, medical comorbidity, fear of surgery, fear of anesthesia and fear of treatment-related side effects [13]. A recent study in the United States on 113,885 patients showed that nearly 19 % of patients with lung/bronchial cancer and non-Hodgkin lymphoma, and more than 16 % of patients with prostate cancer received no treatment for their disease [14]. Not receiving treatment was significantly more common in patients aged >75 years, female patients, in patients from rural areas and patients with an advanced disease stage. 1.1 % of all patients refused treatment that was recommended by their physician. This percentage is an average among all cancer types. Patient refusals of treatment appeared to be related to increasing age, comorbid illness, and lack of perceived clinical benefit. These factors, associated with declining curative treatment, are comparable with the results found in our study. However the average percentage of patients who decline standard treatment is far lower than the 7 % we found and also lower than the frequencies found in other HNSCC studies [10–12]. Hence, it appears that patients with HNSCC have a higher risk of receiving or choosing nonstandard treatment compared to patients with other types of cancer. A study on patients with advanced colon adenocarcinoma did, however, show quite similar results to our study, with a proportion of 18 % of patients that did not receive treatment due to decisions made by their oncologist and 9 % of patients that refused treatment themselves [15]. Older patients were more likely to be recommended nonstandard treatment and were more likely to refuse it, if recommended. Patients living alone and patients with a lot of comorbidity were more likely to receive nonstandard treatment due to the decision by their physician or due to their own choice. This is in agreement with the findings from a breast cancer study, which suggested that older unmarried women were more concerned than married women about treatment-related problems after surgery [16]. A possible factor in the behavior of physicians and patients regarding a choice of therapy is probably poor prognosis.

In our study, overall 3-year survival was lower in patients who received nonstandard treatment. The level of comorbidity was higher and general health status was lower in patients in whom the MDT advised nonstandard treatment. This could be an explanation for the lower survival in these patients [17]. However, there was a significant difference in overall survival between patients who received nonstandard treatment due to a decision made by the multidisciplinary team in relation to patients who refused any type of treatment or declined standard treatment themselves.

When patients or physicians are non-compliant with standard treatment guidelines, for whatever reason, it is not surprising that less curative treatment options, and moreover non curative treatment options will be proposed, both leading to worse survival. Hence, it is still relevant to know to what extent survival differs between these groups of patients, especially when focusing on counselling of patients in whom nonstandard treatment options are considered. How should one approach those patients in daily clinical practice? It is possible that patients who are more accepting of their disease and its prognosis may have treatment goals that differ from those who are not. Improved or preserved quality of life instead of an increased chance of cure and survival could be an explanation for this decisional behavior of patients declining standard treatment options. These findings should be taken into account when counselling patients for whom nonstandard treatment is considered. On the other hand, it is debatable whether these noncompliant patients should be counselled otherwise. Future research should elicit whether the quality of life is improved when patients make more informed choices, independent from what physicians advise.

## Conclusions

Identification of patients with a high risk of receiving nonstandard treatment for curative HNSCC, due to a decision by their physician or themselves, is made possible by this report. Patients living alone, patients with a lot of comorbidity or high tumor stage, females and older patients are more likely to receive nonstandard treatment for curative HNSCC. Therefore we advocate individualized counselling of patients regarding prognosis, quality of life and patient wishes and expectations to achieve shared decision making in treatment for HNSCC.

Our study confirms that the choice of treatment for patients with head and neck cancer should be based on the wishes and motivation of these patients too. In the decision making process, it is important to actively involve the patient and to make sure the patient understands the complexity of the medical problem and the prognosis. Prognostic models based on individual patient characteristics enhance our insight in prognosis of each individual patient. These models can therefore be used in counselling of patients to improve informed decision making [18–20]. We have initiated a prospective trial in our clinic to measure the effect of prognostic counselling using models on treatment outcome, quality of life, patient satisfaction and decisional conflict. In our view, individualized counselling of patients, regarding prognosis, expectations and quality of life, is necessary, before a decision about treatment for HNSCC is made.

## Abbreviations

HNSCC: Head and neck squamous cell carcinoma
ACE27: Adult comorbidity evaluation-27
UICC: Union for international cancer control
MDT: Multidisciplinary tumor board
IKNL: Comprehensive cancer centre the Netherlands
OR: Odds ratio
CI: Confidence interval
